# Psychoeducational intervention and prevention of relapse among schizophrenic disorders in the Italian community psychiatric network

**DOI:** 10.1186/1745-0179-3-7

**Published:** 2007-06-25

**Authors:** Eugenio Aguglia, Elisabetta Pascolo-Fabrici, Francesca Bertossi, Mariano Bassi

**Affiliations:** 1 Psychiatric Clinic of the University of Trieste, Via Paolo De Ralli 5, 34126, Trieste, Italy; 2 Mental Health Department, Local Health Unit, AUSL Via Castiglione 29, 40124 Bologna, Italy

## Abstract

**Background:**

The lack of compliance is associated with an increased risk of hospitalization and switching or augmentation of therapy when compared with being compliant. A synergy of drug therapy and psychosocial interventions can give more benefits in treatment.

**Methods:**

A perspective study was conducted on 150 patients with schizophrenia over 15 centers in Italy. The experimental group was treated with drug therapy, traditional psychosocial and psychoeducation for the patients and their families, while the control group received traditional psychosocial and drug intervention over 1 year.

**Results:**

The experimental group showed a significant statistical improvement (p < 0,05) in almost all the scales that have been assessed (BPRS, SAPS, SANS, SIMPSON-ANGUS SCALE, LANCASHIRE QL SCALE). Significant was the reduction of the number of hospitalizations and of days of hospital stay.

**Conclusion:**

As it is shown in international literature, psychoeducational intervention with schizophrenic patients and their families can reduce the occurrence of relapse.

## 1. Background

The recent changes in the treatment of schizophrenic disorders allow us to use both traditional and atypical antipsychotic drugs, and psychosocial interventions with a reliable efficacy, in treating the symptoms of both positive and negative schizophrenia [1-11].

The clear aim of the treatment of such disorders is not only to control the symptoms, but it is also to prevent new symptomatic acute phases, to bring the patient to comply with the prescribed treatment plan, to restore a certain social and working functioning and to reach a better quality of life.

Among the psychosocial interventions, the psychoeducational ones for the patients and their family, have been considered to be the most promising and successful within the last thirty years [12-14].

The basic principles of the psychoeducational interventions are represented by simple, correct and complete information about the disorder and its possible treatment methods [15].

The goal is also to try to make both the patients and their family aware of those problems, which are related with the disorder, the communication difficulties and the most appropriate management of the stressors and life events.

All of these elements allow the patients and their family to become more conscious and better able to deal with problems, fostering therefore an easier and more effective course of the illness, especially when the psychoeducational interventions are associated with an appropriate and long-term drug treatment [11,16].

A review of those studies published in this field since the beginning of the 1980s, confirms that the use of psychosocial treatments and combined with an appropriate long term antipsychotic therapy, can reduce the percentage of relapse [17] in a year to about 54%. If psychoeducational interventions are carried out with patients and their families [18], in addition to this assertive community approach, the yearly relapses further decrease to 27% [19].

These psychoeducational interventions follow a cognitive-behavioral model. This was probably one of the main causes for the difficult and slow acceptance and popularity of such new therapeutic interventions by the Italian community mental health centers. In fact, still today, despite their overwhelming success rate and their spread among many psychiatric staffs in the world, the psychoeducational approaches are still viewed with suspicion, and many Italian psychiatrists are openly against them. The open and latent fear is accepting therapeutic models which appear to be too simple and limiting, oriented more toward a biological approach of the disorder, which is far from the Italian mental health community and tradition. On the other hand, most of psychiatrist in Italy have adopted a psychodynamic oriented approach, though filtered through the requirements established by social psychiatry and by the "setting" of the public psychiatric services after the Basaglia reform. Often, the idea of introducing new treatment techniques with the most critical patients and their family members is viewed as an attack to already consolidated effective procedures and to rooted cultural models [20].

However, the total effectiveness of the psychoeducational interventions cannot be disputed: in fact more than 20 researches around the world have demonstrated the effectiveness and the encouraging cost-benefit relationship [21-26] for a group of more than 1500 patients [27].

The positive effects of such psychoeducational intervention on the patients and their family, are not only a decrease of new symptomatic acute phases, but also a decrease of the number of hospitalizations and a better compliance with the treatment [27] especially the drug one [1,29-33].

It is generally believed that people affected by schizophrenia that regularly take prescribed antipsychotic drugs, show a faster and more complete remission, and a lower risk of relapse [34].

Since the 1970s, many studies have confirmed that the participation in a long term and stable drug treatment can better prevent relapse compared to a more irregular and discontinuous drug treatment. However, unfortunately, only 50% of people affected by schizophrenia undergo regular an adequate drug treatments for a set period of time [35,36].

On the other hand it is generally agreed that not all patients respond the same to drug treatments and that those treatments do not show the same results with every patient [37].

In many cases the benefits of such drug treatment are only partial and therefore they are not very well liked by the patients and by their families [38].

Several studies have shown that approximately one-third of patients are fully compliant, one-third partially compliant, and the final one third entirely non-compliant. [39-41].

Another study demonstrated that 54,5% of patients were compliant and that 39,0% were partially compliant. Partial compliance was associated with an increased risk of hospitalization and switching or augmentation of therapy when compared with being compliant [42].

A decrease in compliance predicted an increase in PANSS which corresponds to a worsening of symptoms [43].

A low compliance also predicts an increase risk of hospitalization: even small gaps in therapy (1–10 days) increased the likelihood of hospitalization by twice, whereas larger gaps in therapy (>30 days) increased the likelihood of hospitalization by four times [43].

The psychoeducational interventions can facilitate schizophrenic patients in gaining the necessary skills to effectively manage a drug treatment.

In order to have the patient comply to an antipsychotic drug treatment in a more appropriate way, it is necessary to: 1) assess thoroughly the patient's background in terms of past drug therapies and of those factors which might have prevented the compliance; 2) use, whenever it is possible, a "contractual" approach, in which any potential change from its original layout can easily be discussed with the patient; 3) educate the patients and the family members on the disorder and its characteristics, and on the prevention of potential relapses, making them aware of the risks and the benefits of antipsychotic drugs; 4) maintain, whenever it is possible, the control over those patients who may temporarily neglect and/or refuse the drug treatment, still offering them alternative solutions [1,44].

Informative, short term psychoeducational interventions seem to be not as effective in the long run, in maintaining the compliance[28,45-47].

On the other hand, a more structured and prolonged psychoeducational treatment for patients and their families, seem to be more effective in the long run [4,10,48-53].

## 2. Methods

### 2.1 Design of the study

This study was conducted in Italy, with the goal of trying to identify the most effective tools in the prevention of relapse among those affected by schizophrenia.

The main objective of the study was to assess the effectiveness of the combination of a long term drug therapy and a psychoeducational intervention, on people affected by schizophrenia in reducing relapses in terms of number of hospitalisations and clinical parameters.

The Italian protocol was developed based on the model of a study conducted in Munich between 1990 and 1994, by Kissling and Bauml, with a sample of 236 patients [1,54].

In this study, half of the patients affected by schizophrenia underwent a traditional drug treatment (the control group), while the second half of the sample underwent a treatment, which included also, along with the traditional psychosocial interventions, a psychoeducational treatment. Traditional treatment together with psychoeducation was able to reduce of 45% the number of hospitalization [1,54].

In Italy a an open, controlled multicentric research was conducted in 15 Italian Community Mental Health Centers (Public Mental Health Departments and University Psychiatric Clinics), for the duration of 1 year, excluding the screening phases. In each CMHC, after each screening phase, the patients were blindly randomized by the experimenter into two groups. The control group (of a total of 66 people) was treated with a standard procedure (antipsychotic drug treatment and assertive community treatment), while the study group (69 patients) received, traditional psychosocial intervention, antipsychotic drug treatment and a psychoeducational program. Such sample, comprised of patients and their families, participated separately to 8 different parallel psychoeducational meetings, of 60–90 minutes each. Such meetings were characterized by an overlapping informative content, and were run by two psychiatric operators (mainly by a psychiatrist and a psychiatric nurse).

The scales assessment was carried out at the beginning of the trial, after 6 months (T2), and 12 months (T4). At the end of the study the parameters "Number of hospitalizations" and "Total number of hospital days" were checked. Each time drug recording and vital parameters were assessed. (Tab 1, Research protocol)

**Table 1:** Research protocol

*Time T-x*	First screening phase
***Time T 0***	- Vital parameters
	- Analysis of the drug treatment characteristics and Drug treatment registration
	- Assessment scales administration
***Time T 1 ***(After 3 months)	- Vital parameters
***Time T 2 ***(After 6 months)	- Drug treatment registration
	- Assessment scales administration
***Time T 3 ***(After 9 months)	- Vital parameters
***Time T 4 ***(After 12 months)	- Vital parameters
	- Assessment scales administration
	- Control for the re-hospitalization rate (Number of hospitalizations, Total number of hospital days)

### 2.2 Patients

150 patients took part in this study. Their age ranged from 18 and 45 years. They were all diagnosed with schizophrenia, in agreement with the DSM IV (Diagnostic and Statistical Manual of Mental Disorders, fourth edition) and the ICD 10 (International Classification of Diseases, tenth edition) and were undergoing a standardized therapy in terms of types of drugs and dosages.

A set of criteria for the exclusion from the study included: acute psychosis, a substance abuse problem, organic factors that could interfere with the clinical condition, the patients' current participation in psychoeducational and structured treatments, or their participation in the last two years.

The analysis of the participants has shown no significant clinical and socio-demographic differences between the two groups. 135 patients finished the study; 15 dropped out for different reasons unrelated to the study.

### 2.3 Antipsychotic drug treatment

Both groups followed the antipsychotic drug treatment using traditional and atypical antipsychotic drugs, administered alone or combined. The current dosage was monitored every 6 months, from the beginning of the study. An equal percentage of patients in both the control and the study groups, received also, during the study, some depot or "long acting" (haloperidol decanoate, fluphenazine decanoate, zuclopentixol decanoate) anti-psychotic drugs.

### 2.4 Psychiatric assessment Scales

The following scales were administered during the study: BPRS (Brief Psychiatric Rating Scale), SAPS (Scale for Assessment of Positive Symptoms), SANS (Scale for Assessment of Negative Symptoms), Sympson and Angus Scale, ROMI (Rating of Medication Influences) and the Lancaster QL (Lancaster Quality of Life Profile).

### 2.5 Psychoeducational program

The standardized psychoeducational program, managed through an interactive educational method, took place in 8 sessions, in which the following topics were covered:

1. Introduction

2. What is schizophrenia?

3. What causes schizophrenia?

4. How to treat schizophrenia?

5. Psychosocial treatment strategies

6. Preventing relapses

7. The role of the family

8. Conclusion

### 2.6 Statistical methods

All of the data was elaborated and statistically measured using the SAS procedure v 6.12. The basal homogeneity of the groups was measured for both the demographic characteristics and the measuring scales, using the Wilcoxon's non-parametric test.

The measurement within treatments was conducted taking in consideration the starting and ending time of the treatment: the significance of the difference was measured using the Sign Rank Test.

The treatments were compared using an ANOVA model, in which the fixed effects were the treatment and the centers; the assessment of the differences was done on the LS means (means calculated with the least squares' method) using the LSD test of the PRCO GLM.

### 2.7 Consent

The study was approved by the ethical committee and the patient gave written informed consent.

## 3. Results

### 3.1 Socio demographic and clinical parameters

**Figure 1:**
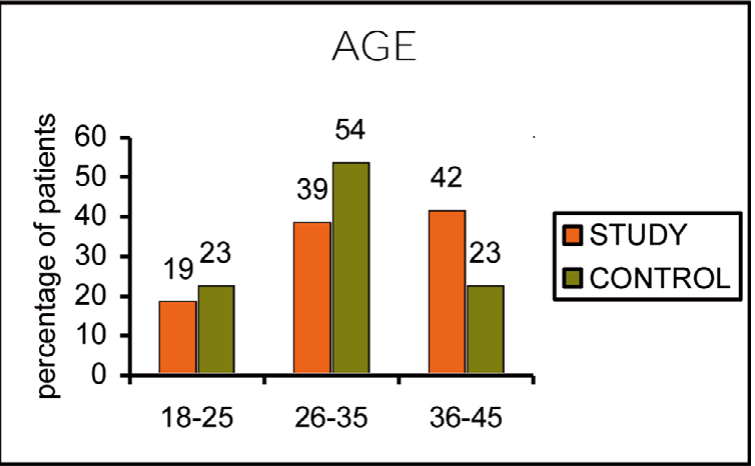
Age.

**Figure 2:**
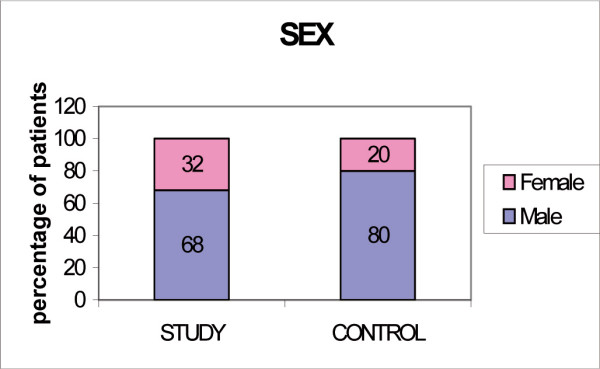
Sex.

The two subgroups of subjects participating in the test have the same clinical and socio-demographic characteristics. The percentage of male vs female and the age groups stratification in both groups appears to be similar, and the differences are not of statistical significance. (Fig 1, Age ; Fig 2, Sex)

Heart rate, blood pressure and body weight measurements have remained the same during the entire treatment program. (Tab. 2, Vital parameters)

**Table 2:** Vital parameters

(p > 0.05) VITAL PARAMETERS	T0	T1	T2	T3	T4
Heart rate (Beats/min)					
STUDY	81,92	80,58	81,80	80,55	80,97
CONTROL	79,92	78,82	80,02	80,64	82,16
Systolic pressure (mmHg)					
STUDY	119,90	118,72	120,43	121,97	121,50
CONTROL	121,35	120,54	121,91	120,33	122,97
Diastolic pressure (mmHg)					
STUDY	75,73	75,09	75,33	75,97	76,33
CONTROL	75,63	75,74	75,53	74,66	77,33
Body weight (Kg)					
STUDY	76,04	76,52	76,78	77,26	75,90
CONTROL	76,98	77,70	76,63	78,01	79,65

Diagnosis according to the DSM IV has brought to a further subdivision of the different subtypes of schizophrenia, the main one being the paranoid type, respectively 40% in the study group and 43% in the control group (difference n.s.). (Tab. 3, Diagnosis)

**Table 3:** Diagnosis

DIAGNOSIS		
	STUDY (%)	CONTROL (%)
Schizophrenia	15	17
Disorganized Schizophrenia	9	23
Paranoid Schizophrenia	40	43
Catatonic Schizophrenia	2	0
Non-differentiated Schizophrenia	19	13
Residual Schizophrenia	15	4

Time from the first diagnosis, the years of treatment, the number of hospitalizations, and the current psychopharmacological treatment, were also kept under consideration.

All of the parameters have resulted substantially homogeneous and there was no substantial statistical significance between the two study groups. (Tab. 4, Clinical parameters: years from the diagnosis, years of treatment, number of hospitalizations)

**Table 4:** Clinical parameters: years from the diagnosis, years of treatment, number of hospitalizations

Clinical parameters	Duration	STUDY (%)	CONTROL (%)
Number of years from the diagnosis of schizophrenia	Less than 1 year	2	4
	From 1 to 5 years	31	55
	From 6 to 10 years	37	19
	Over 10 years	30	22
Number of years of treatment	Less than l year	5	5
	From 1 to 5 years	36	56
	From 6 to 10 years	34	19
	Over 10 years	25	20
Number of hospitalizations in the last three years	None	28	26
	From 1 to 3	48	47
	From 4 to 6	18	19
	From 7 to 10	2	6
	Over 10	4	2

### 3.2 Assessment Scales

**Figure 3:**
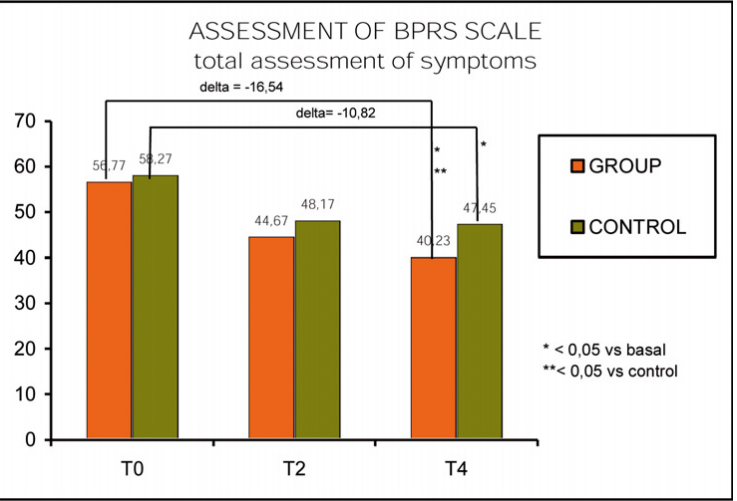
BPRS scale.

The BPRS showed a decrease in the gravity of symptoms for both groups, but while the control group varied from a basic score of 58.27 to a score of 47.45 after 12 months with an improvement of 10.82 points (p < 0.05). The study group showed a score of 56.77 at T0 and of 40.23 at T4 with 16.54 points of difference (p < 0.05). The difference between the groups was statistically significant in favor to the study group. (p < 0.05). (Fig. 3, BPRS scale)

The detailed analysis of the single items of the BPRS scale, allows to highlight the items that changed most. The study group has shown a better improvement (p < 0.05) in delayed

emotional flattening, anxiety, emotional withdrawal, distractibility, thought disorganization, lack of collaboration, artificial attitude. The motor skill and the excitability items decreased equally in both groups (p > 0.05) (Tab. 5, BPRS scale: assessment of item "emotion").

**Table 5:** BPRS scale: assessment of item "emotion"

THE BPRS SCALE Assessment of each item: EMOTION						
	GROUP			CONTOL		

	T0	T4	*Diff*.	T0	T4	*Diff*,

Thought disorganization	2,56	2,00	*-0,56*	2,34	2,18	*-0,16*
Emotional flattening	3,24	2,24	*-7,00*	2,80	2,61	*-0,79*
Emotional withdrawal	3,37	2,34	*-7,03*	3,18	2,80	*-0,38*
Delayed motor skills	2,53	1,42	*-1,11*	2,13	1,85	*-0,28*
Tension	3,48	2,16	*-1.32*	3,10	2,55	*-0,55*
Lack of compliance	2,11	1,29	*-0,82*	1,98	1,56	*-0,42*
Excitement	1,63	1,11	*-0,52*	1,99	1,44	*-0,55*
Distractibility	2,38	1,40	*-0.98*	2,30	1,80	*-0,50*
Motor skill hyperactivity	1,59	1,06	*-0,53*	1,76	1,32	*-0,44*
Artificial behavior	1,89	1,24	*-0,65*	1,92	1,67	*-0,25*
TOTAL Assessment	24,80	16,27	*-8,53*	23,47	19,77	*-3,70*

The study group had a significant improvement (p < 0.05) in items of the BPRS Behaviour subscale such as: depression, personal neglect, somatic preoccupation, and unusual thought process. (Tab. 6, BPRS scale: assessment of item: "behavior")

**Table 6:** BPRS scale: assessment of item: "behaviour"

BPRS SCALE Assessment of each item: BEHAVIOUR						
	GROUP			CONTROL		

	T0	T4	*Diff*,	T0	T4	*Diff*.

Somatic preoccupation	3,23	2,08	*-1,15*	2,77	2,23	*-0,54*
Anxiety	3,59	2,40	*-1,19*	3,25	2,47	*-0,78*
Depression	2,71	1,53	*-1,18*	2,52	2,08	*-0,44*
Suicidal tendencies	1,01	0,85	*-0.16*	1,11	1,03	*-0,08*
Feelings of guilt	2,03	1,21	*-0,82*	1,71	1,60	*-0,11*
Hostility	2,17	1,32	*-0,85*	2,48	1,73	*-0,75*
Over excitement	1,61	1.06	*-0,55*	1,76	1,36	*-0,40*
Grandiosity	1,99	1,26	*-0,73*	2,18	1,59	*-0,59*
Suspicious behavior	2,94	1,76	*-0,18*	3,67	2,48	*-0,19*
Hallucinations	2,50	1,58	*-0,92*	2,55	1,91	*-0,64*
Unusual thought process	3,59	2,27	*-1,32*	3.23	2,45	*-0,78*
Strange behavior	2,50	1,71	*-0,79*	2,72	2,08	*-0,64*
Personal neglect	2,48	1,53	*-0,95*	2,32	2,09	*-0,23*
Disorientation	1,47	1,00	*-0,47*	1,13	1,27	*0,74*
TOTAL Assessment	33,82	21,56	*-12,26*	33,26	26,24	*-7,02*

**Figure 4:**
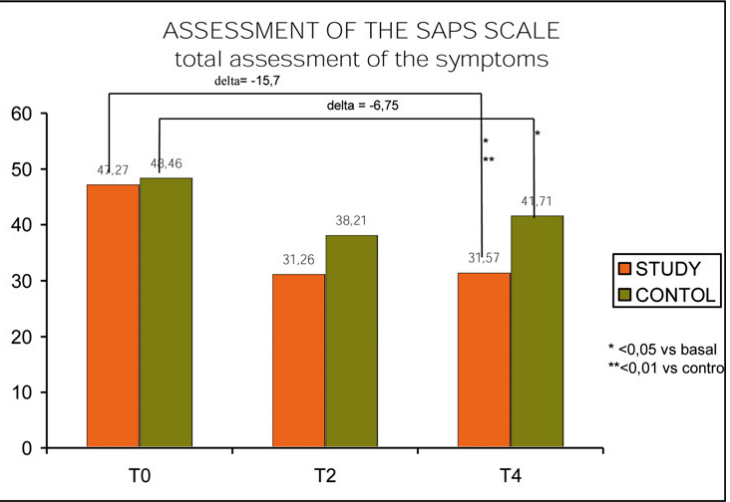
SAPS scale.

SAPS Scale was used to asses positive symptoms (hallucinations, delusions, strange behavior, thought disorder). The total score has shown a statistical significant decrease for both groups. The study group changed from 47.2 to 32.57 points with a difference of 15.7 points (<0.05); the control group varied from 48.46 to 41.71 points with a 6.75 point difference. A highly significant difference was found between the two groups (<0.001). (Fig. 4, SAPS scale)

**Figure 5:**
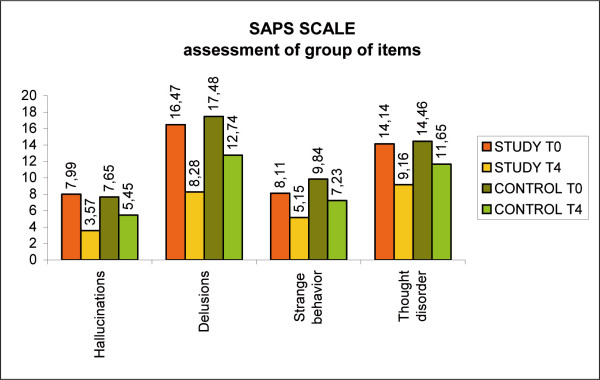
SAPS scale, group of items.

A reduction in the delusional symptoms has contributed to a significant difference of 8.19 points compared with 4.74 points for the control group (p < 0.01). (Fig. 5, SAPS scale, group of items)

**Figure 6:**
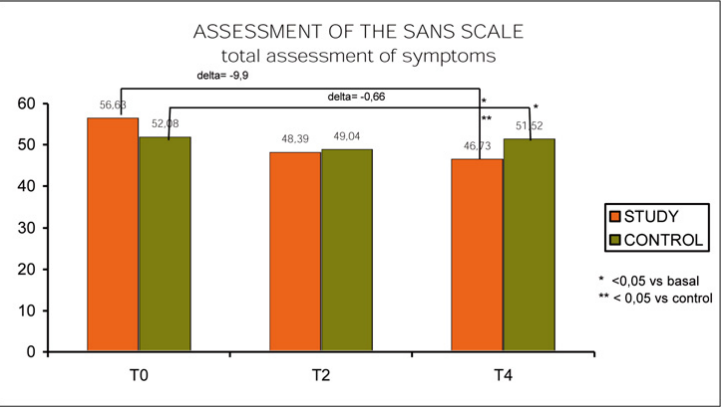
SANS scale.

The analysis of the changes in the negative symptoms was conducted using the SANS Scale. The study group showed an improvement of 9.9 points (from 56.63, to 46.73 points, p < 0.05) while the control group had and improvement of only 0.66 points, going from 52.8 to 51.52. (n.s). The difference between the groups had statistical significance (p < 0.05) (Fig. 6, SANS scale).

**Figure 7:**
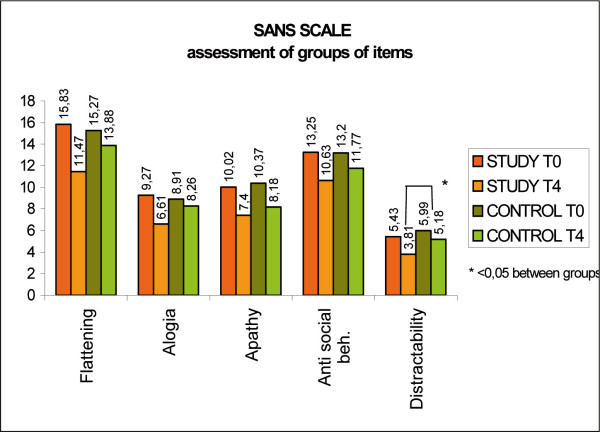
SANS scale, group of items.

The study group showed a significant difference in the item distraction, showing a change of 1.62 points compared to the 0.81 points of change for the control group (p < 0.05). As for the other parameters, the study group scored better improvements, but the difference between the groups was non significant (p > 0.05). (Fig 7, SANS scale, group of items)

The ROMI Scale measures the reasons for participating or not in a treatment program and was assessed by both the patients and the research physicians.

**Figure 8:**
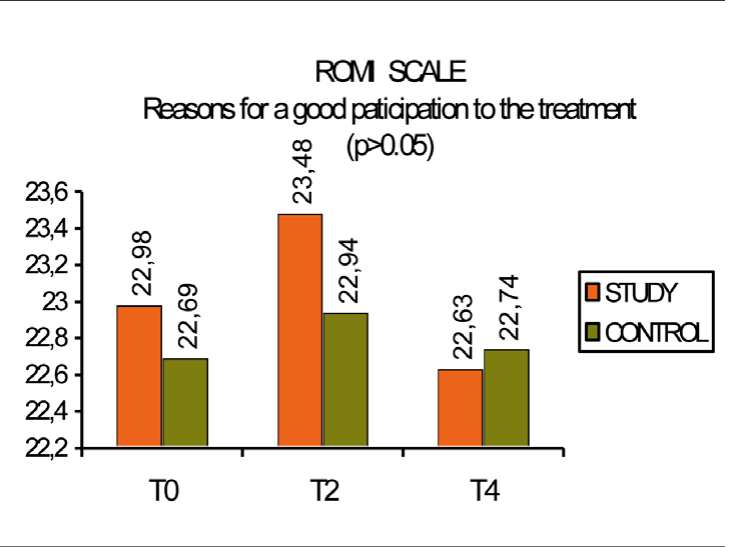
ROMI scale: reasons for participating.

The subset "Reasons for good participating to the treatment", showed no difference between the groups (p > 0.05). (Fig 8, ROMI scale: reasons for participating)

**Figure 9:**
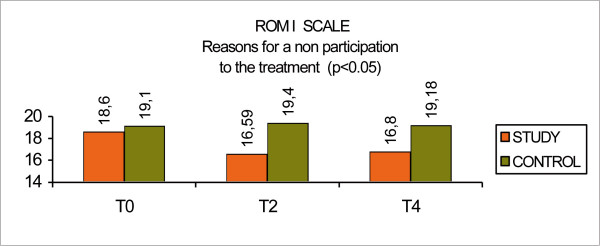
ROMI scale: reasons for non participating.

The subset "Reasons for non participating to the treatment" such as bad relationship between the doctor and the patient, bad relationship with the psychiatric staff, denial about the disease, need for current treatment, desire for hospitalization, interference with personal activities, refusal to take medications, were improved in the control group with and increase of 0.08 points compared to a decrease of 1.8 points for the study group (p < 0.05). (Fig 9, ROMI scale: reasons for non participating)

**Figure 10:**
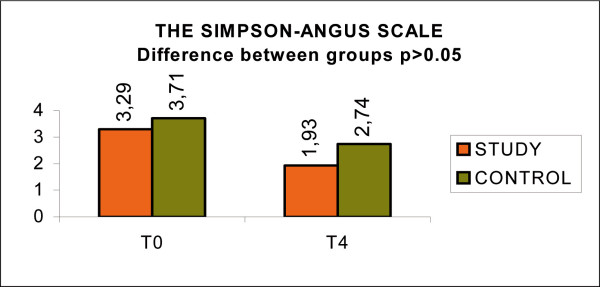
Simpson-Angus scale.

The Simpson-Angus Scale measures the assessment of the extrapyramidal effects. Though the different treatments were comparable between the two groups, the study group showed a decrease of 1.36 points compared with 0.97 points for the control group but the difference was non significant.(p > 0.05). (Fig. 10, Simpson-Angus scale)

**Figure 11:**
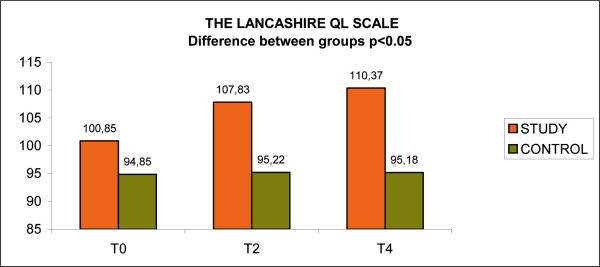
Lancashire QL scale.

The quality of life at the Lancashire QL scale demonstrated ad increase for the study group of 9.52 points (from 100,85 points, to 110,37 points), and an improvement for the control group of 0.33 points for the total scale (p < 0.05); in particular regard to the items wellness, work, leisure, religion, economic situation, family relationship, social relationship, overall wellbeing (p < 0.05). (Fig 11, Lancashire QL scale)

**Figure 12:**
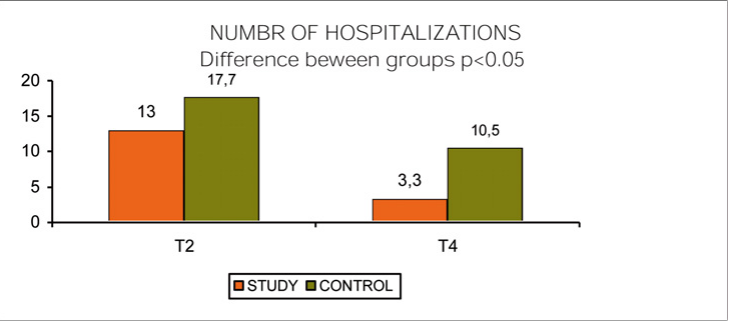
Number of Hospitalizations.

The percentage of the subjects hospitalized between 1 and 3 times during the 12 months resulted to be 13% after 6 months, and 3,3% after a year with a difference of 9.7% for the study group; while for the control group the variation went from 17.7% at 6 months to 10.5% after 1 year with an improvement of 7,2%. The difference between groups showed statistical significance (p < 0.05). (Fig. 12, Number of Hospitalizations)

**Figure 13:**
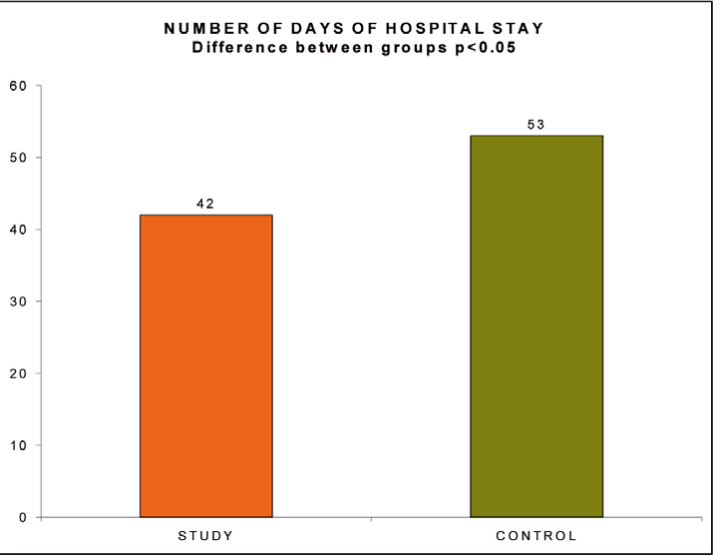
Number of days of hospital stays.

There was also a decrease of mean number of days of hospital stays for each hospitalisationfor the study group (42 days) compared to the control group (53 days); (p < 0.05). (Fig. 13, Number of days of hospital stays)

## 4. Discussion

### 4.1 Findings

The experimental group showed a significant statistical improvement (p < 0,05) in almost all the scales that have been assessed (BPRS, SAPS, SANS, SIMPSON-ANGUS SCALE, LANCASHIRE QL SCALE). Significant was the reduction of relapse in terms of numbers of hospitalization, days of hospital stay and clinical parameters. This was the main objective of our study and was significantly confirmed in the effect of the overall improvent on most clinical parameters, quality of life, relation with the staff.

The different changing in BPRS over the time can suggest the role of psychoeducation in the improvement of clinical parameters. Both groups showed a decrease of gravity of the symptoms in the first 4 months, this can be due to the consolidated synergy between drug treatment and standard psychosocial intervention. The study group keeps on improving over the 12 months of the study and this can be related to the capacity of psychoeducation to help in handling with the symptoms, have a better therapeutic alliance and prevent the relapse of the psychosis. The analysis of BPRS can show how much psychoeducation can help in reduce certain symptoms as emotional flattening, anxiety, emotional withdrawal, distractibility, thought disorganization, lack of collaboration, artificial attitude, but especially we found a particular improvement in depression, personal neglect, somatic preoccupation, and unusual thought process.

To a less extent psychoeducation can improve anxiety, confusion, hallucinations, strange behavior, over excitement, grandiose feelings, hostility, suicidal tendencies. As for the suspicion item, the difference was identical for both groups: a more specific treatment can be required to improve this item.

The changing in both positive and negative symptoms measured by SAPS and SANS scales evidences how psychoeducation can give a generalized improvement over all kind of symptoms.

The changes in ROMI scale highlighted the importance of psychoeducation as shown by the improvement of the relationship with the staff, the increase of awareness of the illness and the needing for treatment, the positive believe of the family, the prevention of relapse and the improvement of compliance. The psychoeducational approach has helped family members to live with patients and their disorder, and, at the same time, it has highlighted the more positive qualities of patients.

The lack of significant difference in the extrapiramidal effect (Simpson-Angus scale) can be explained with the consideration the this parameter is due to the pharmacotherapy and it is not significantly influenced by psychoeducation, though the difference registered between the two groups can show that psychoeducation can teach the patient and the family to recognize earlier the side effects and relate to the doctor for changing the drug treatment.

The study group has shown an important and persistent improvement in the quality of life (Lancashire scale), while the other group had not improved over the year of the study: this difference in due to the overall benefits of psychoeducation that lead to a better adherence to the whole program.

The number of psychoeducational sessions was only 8 and we suggest this program could be further improved in terms of number of sessions, items of discussions, role of the family and the patients in the process in order to better maintain the positive outcomes and the parameters that have not improved.

### 4.3 Methodological issues

This study has some limitations. It is a cross-sectional study and cohort effect can distort the results. The number of the patients in small, so larger studies is needed to confirm this data. This bias could be reduced comparing the data with an international meta-analysis.

Though the staff carrying out psychoeducation and standard psychosocial intervention and the staff administering the scales were different, the open label design of the study could bring some biases. A double blind study should be recommended.

This study has several strengths. It evaluates the efficacy of psychoeducation in the real clinical practice following a multicenter design and it analyses not only the clinical parameters, the relationship and the quality of life as different parameters, but it also focuses on how the improvement of compliance can lead to a reduction of relapse, as it is shown in international literature.

## 5. Conclusion

A number of studies have show that a psychoeducational intervention with schizophrenic patients and their families, could reduce the occurrence of relapse [2,55-58].

Such decrease seems to be related with a decrease in hospitalizations, with less sick days on the job, and with less social expenses [59-61].

The study is part of an international program whose effort is to assess the effectiveness of a psychoeducational treatment in the prevention of relapses, through a change in the understanding and the acceptance of the disorder. Our study confirms improvements on most clinical parameters, quality of life, adherence to the treatment program, reduction of relapse and number of hospitalisation.

A psychoeducational therapeutic approach always seems to have positive effects on both the patients and their family. In fact, results show that even short term educational-informative contents were able to improve the patients' level of compliance to the treatment program, the patients' and their family members' attitude toward the disorder, and their attitude toward the psychiatric staff. Also, such educational approach seem to be able to improve the individuals' perception of quality of life, which represents an indirect tool used to reduce self and hetero-stigmatization. The participation of family members in the study has allowed a more effective management of the patient and a better implementation of a psychosocial rehabilitation process.

In addition to the unquestionable advantages of such integrative treatment plan, there is also a decrease in costs associated with hospitalizations, loss of working days, tension, family and social apprehensions.

In conclusion, such multicentric experience allows us to confirm that the psychoeducational approach has contributed significantly to an integrated approach that put together patients active role in managing symptoms, family members participation and psychiatric staff work that led to a global improvement and a reduction of relapses and hospitalizations.

The result is an undoubtedly great advantage for the patients, who becomes an active participant in their therapeutic process, experiencing not only an improvement from a clinical stand point, but also an overall increased psychological wellness, reducing significantly the troubles and the bad feelings caused by such afflicting disorder.

## Competing interests

The author(s) declare that they have no competing interests.
